# Prenatal Household Air Pollution Exposure, Cord Blood Mononuclear Cell Telomere Length and Age Four Blood Pressure: Evidence from a Ghanaian Pregnancy Cohort

**DOI:** 10.3390/toxics9070169

**Published:** 2021-07-14

**Authors:** Seyram Kaali, Darby Jack, Jones Opoku-Mensah, Tessa Bloomquist, Joseph Aanaro, Ashlinn Quinn, Ellen Abrafi Boamah-Kaali, Patrick Kinney, Mohammed Nuhu Mujtaba, Oscar Agyei, Abena Konadu Yawson, Samuel Osei-Owusu, Rupert Delimini, Blair Wylie, Kenneth Ayuurebobi Ae-Ngibise, Andrea Baccarelli, Seth Owusu-Agyei, Steven N. Chillrud, Kwaku Poku Asante, Alison Lee

**Affiliations:** 1 Kintampo Health Research Centre, Ghana Health Service, Kintampo P.O. Box 200, Bono East Region, Ghana; jones.opoku-mensah@kintampo-hrc.org (J.O.-M.); janaaro02@gmail.com (J.A.); ellen.boamah@kintampo-hrc.org (E.A.B.-K.); mohammed.mujtaba@kintampo-hrc.org (M.N.M.); oscar.agyei@kintampo-hrc.org (O.A.); abenayawson@hotmail.com (A.K.Y.); samuel.osei-owusu@kintampo-hrc.org (S.O.-O.); kenneth.asayah@kintampo-hrc.org (K.A.A.-N.); seth.owusuagyei@gmail.com (S.O.-A.); kwakupoku.asante@kintampo-hrc.org (K.P.A.); 2 Department of Environmental Health Sciences, Mailman School of Public Health, Columbia University, New York, NY 10032, USA; dj2183@cumc.columbia.edu (D.J.); tb2715@cumc.columbia.edu (T.B.); ab4303@cumc.columbia.edu (A.B.); 3 Fogarty International Centre, National Institute of Health, Bethesda, MD 20892, USA; aquinn@berkeleyair.com; 4 Department of Environmental Health, Boston University School of Public Health, Boston, MA 02118, USA; pkinney@bu.edu; 5 Department of Biomedical Sciences, University of Health and Allied Sciences, Ho PMB 31, Volta Region, Ghana; rdelimini@uhas.edu.gh; 6 Beth Israel Deaconess Medical Centre, Division of Maternal-Fetal Medicine, Department of Obstetrics and Gynecology, Boston, MA 02215, USA; bwylie@bidmc.harvard.edu; 7 Institute of Health Research, University of Health and Allied Sciences, Ho PMB 31, Volta Region, Ghana; 8 Department, Lamont Doherty Earth Observatory at Columbia University, Palisades, NY 10964, USA; chilli@ldeo.columbia.edu; 9 Division of Pulmonary, Critical Care and Sleep Medicine, Icahn School of Medicine at Mount Sinai, New York, NY 10029, USA; Alison.Lee@mssm.edu

**Keywords:** prenatal household air pollution, oxidative stress, telomere length, childhood blood pressure, sex-specific effects, cardiovascular disease

## Abstract

Associations between prenatal household air pollution exposure (HAP), newborn telomere length and early childhood blood pressure are unknown. Methods: Pregnant women were randomized to liquefied petroleum gas (LPG) stove, improved biomass stove or control (traditional, open fire cook stove). HAP was measured by personal carbon monoxide (CO) (*n* = 97) and fine particulate matter (PM_2.5_) (*n* = 60). At birth, cord blood mononuclear cells (CBMCs) were collected for telomere length (TL) analyses. At child age four years, we measured resting blood pressure (BP) (*n* = 97). We employed multivariable linear regression to determine associations between prenatal HAP and cookstove arm and assessed CBMC relative to TL separately. We then examined associations between CBMC TL and resting BP. Results: Higher prenatal PM_2.5_ exposure was associated with reduced TL (β = −4.9% (95% CI −8.6, −0.4), *p* = 0.03, per 10 ug/m^3^ increase in PM_2.5_). Infants born to mothers randomized to the LPG cookstove had longer TL (β = 55.3% (95% CI 16.2, 109.6), *p* < 0.01)) compared with control. In all children, shorter TL was associated with higher systolic BP (SBP) (β = 0.35 mmHg (95% CI 0.001, 0.71), *p* = 0.05, per 10% decrease in TL). Increased prenatal HAP exposure is associated with shorter TL at birth. Shorter TL at birth is associated with higher age four BP, suggesting that TL at birth may be a biomarker of HAP-associated disease risk.

## 1. Introduction

Over 40% of the world’s population is exposed daily to household air pollution (HAP) from the burning of solid fuels in combustion-inefficient stoves [1]. HAP results in 2.3 million premature deaths and 91.5 million disability adjusted life years (DALYs) annually [2], mostly in low- and middle-income countries (LMICs) [3], with the largest burden of disease—approximately 46% of mortality and 36% of DALYs—attributable to cardiovascular disease [4]. Women commonly are the primary cooks and continue to cook while pregnant; thus, exposure to HAP begins in utero. Epidemiological data suggest that oxidative stress (OS) is central to air pollution pathogenesis. Maternal air pollution exposures may induce fetal OS directly from translocation of fine particulate matter with aerodynamic diameter <2.5 µm (PM_2.5_) across the placenta [5] or indirectly through induction of maternal OS, with implications for development and future health [6,7]. Our group has previously demonstrated that increased prenatal HAP exposure is associated with reduced cord blood mitochondrial DNA copy number, a biomarker of increased OS [8].

Telomeres are repeating DNA segments at chromosome ends that regulate cellular function and protect against chromosomal degradation [9,10]. Telomeres may shorten with each cell cycle, and shortening may be accelerated by pro-oxidant environmental exposures. Cellular senescence is induced when cells reach a specific number of divisions, or the Hayflick limit. Importantly, telomerase expression can override the Hayflick limit [11], implicating telomeres in cellular senescence. Telomere length (TL) at birth and subsequent rate of attrition are major determinants of TL in adulthood [12,13], which has been associated with chronic diseases including cardiovascular diseases such as stroke and coronary artery disease and chronic respiratory disease [14,15,16,17]. The prenatal period may be a time of TL plasticity; enzymes that prevent telomere shortening have reduced activity late in gestation, thus increasing vulnerability to shortening [18]. Taken together, these data suggest that TL at birth may be a biomarker of prenatal environmental risk.

Currently there is a lack of evidence on the associations between prenatal HAP exposure and TL at birth. Supporting evidence suggests that prenatal ambient air pollution exposures may alter TL at birth, although results have been conflicting. For example, data from the Mexico City Programming Research in Obesity, Growth, Environment and Social Stressors (PROGRESS) cohort and the European (Environmental Influences on Early Ageing) ENVIR*ON*AGE cohort found that increased PM_2.5_ exposure in early or mid-gestation was associated with shorter TL, while increased exposure in later pregnancy was associated with longer TL [19,20]. Data from the New York City (Programming of Intergenerational Stress Mechanisms (PRISM) cohort found that increased PM exposure in mid-gestation alone shortened TL [21], while data from a Chinese birth cohort suggested that exposure in later pregnancy shortened TL [22]. As sex appears to influence TL, with longer telomeres described in females as compared with age-matched males [23,24], these studies further explored sex-specific effects. Results were conflicting, with one study finding girls were more vulnerable [19], two finding boys were more vulnerable [21,22] and one finding no sex-specific effects [20]. To our knowledge, only two studies have examined the effect of HAP exposure on TL. Both studies were in adults and found an inverse association between HAP and TL [25,26]. 

Exposure to air pollution and specifically PM_2.5_ is one of the most important environmental risk factors for the development of cardiovascular disease (CVD) [27]. Early life, including the prenatal period, represents a critical period of developmental plasticity when cardiovascular health set-points and trajectories are determined [28,29]. Epidemiologic evidence suggests that BP tracks over the life course [29,30] and that markers of cardiovascular health in childhood correlate with risk for future CVD [31,32,33]. In adults, elevated blood pressure is a major risk factor for CVD and accounts for 13% of all deaths worldwide including 51% of stroke deaths and 45% of coronary heart disease deaths [34]. In situ atherosclerosis studies of vascular smooth muscle and endothelial cells demonstrate the role of telomeres in cellular senescence [35]. A meta-analysis of 24 studies with >43,000 adult participants found that shorter leukocyte TL was associated with increased risk for coronary heart disease (non-fatal myocardial infarction, coronary heart disease death or coronary revascularization; pooled relative risk 1.54, 95% CI 1.30, 1.83) and cerebrovascular disease (non-fatal stroke or death from cerebrovascular disease; pooled relative risk 1.42, 95% CI 1.11, 1.81) [16]. To our knowledge, these observations have not been extended to TL at birth and markers of cardiovascular health in early childhood such as BP. Identification of biomarkers of environmental risk that are associated with future cardiovascular health may allow identification of at-risk children for public health interventions.

We leveraged a Ghanaian pregnancy cohort derived from the Ghana Randomized Air Pollution and Health Study (GRAPHS) to examine associations between prenatal HAP exposure, newborn TL and resting BP at child age four years. Specifically, we first examined exposure–response relationships between prenatal HAP exposure and TL at birth. We also explored associations between specific cookstove interventions and TL at birth. Then we examined associations between TL at birth and resting BP at child age four years. Our limited sample precluded a formal mediation analysis of TL as a mediator of the association between HAP and BP.

## 2. Materials and Methods

### 2.1. Study Participants 

Participants were from a Ghanaian pregnancy cohort derived from GRAPHS, a cluster randomized stove intervention trial in the Kintampo North Municipality and Kintampo South District of Ghana [36]. As has been described elsewhere [36], GRAPHS recruited *n* = 1414 non-smoking, pregnant women at an ultrasound-confirmed gestational age ≤24 weeks between August 2013 and March 2016. Eligible women were randomized to one of two stove interventions, a liquefied petroleum gas (LPG) stove, an improved combustion-efficiency biomass stove (BioLite Stove) or a traditional, open-fire stove (control). Prenatal HAP exposures were quantified by repeated maternal personal exposure assessments. GRAPHS concluded when study children reached one year of age. A subset of children (*n* = 700) were prospectively followed up to age 7 years to better characterize child health, including cardiovascular health. 

A nested study from October 2014 to August 2015 consecutively recruited *n* = 157 GRAPHS pregnant women for cord blood collection. Only women who delivered in a clinic were included in this substudy. Cord blood was drawn immediately following delivery, and cord blood mononuclear cell (CBMC) was isolated with subsequent DNA extraction and TL analysis. Of these, *n* = 97 mother–infant dyads were included in the ongoing prospective cohort with available age four resting blood pressure data. Laboratory analysis of TL was blinded to cookstove assignment. Similarly, staff who measured blood pressure at age four were not aware of TL results. The study was approved by the Kintampo Health Research Centre’s Institutional Ethics Committee in Ghana, and the Institutional Review Boards of the Icahn School of Medicine at Mount Sinai and Columbia University Mailman School of Public Health in the United States. Written informed consent was obtained from all mothers prior to commencement of study activities.

### 2.2. Prenatal CO and PM_2.5_ Measurements 

We indexed prenatal HAP exposure by 72-h personal CO and PM_2.5_ assessments. Pregnant women performed 72-h personal CO assessments once prior to randomization and three times equally spaced between stove assignment and ultrasound-estimated date of delivery (Lascar EL-CO-USB Data Logger, Essex, UK). As was done in prior analyses, linear interpolation of CO values was used to create a time-weighted average across gestation [37]. In a subset of participants, and at one prenatal time point following stove assignment, we concurrently measured 72-h personal PM_2.5_ exposure (RTI microPEM, Research Triangle Park, NC, USA) co-located on the participant with the CO monitor. The personal monitors were affixed to participants’ clothing near their breathing zone, and participants were asked to only remove the monitors at bedtime or during bathing. Details of exposure measurement and quality control measures have been reported elsewhere [36,37,38]. Briefly, key aspects of the microPEM is that it includes a two-stage impactor inlet that only allows particles with mean aerodynamic diameter less than 2.5 µm to pass into the sensing chamber of the nephelometer that provides continuous measurements, after which the PM_2.5_ is collected onto a Teflon filter, allowing the average response of the nephelometer to be calibrated against the net weight of the particulate matter. To achieve target runtime, the microPEM was run with a 50% duty cycle (30 s on/30 s off); during each on cycle, 3 nephelometer readings were recorded, which were then averaged to provide minute average response. To assess and correct for baseline drift, a HEPA filter was attached in the field at the beginning and end of each 72 h deployment. Data were not retained if the pre- and post-deployment HEPA periods were outside of acceptable range (±20 µg/m^3^). This HEPA adjustment to all the data was done before gravimetrically correcting the average nephelometer response to the gravimetrically determined average PM_2.5_ concentration.

The Lascar monitors recorded CO in parts per million (ppm) every 10 seconds. Data used in these analyses passed three quality assurance/quality control checks, including: exposing monitors to certified span gas (50 ppm CO in zero air) at the KHRC lab every 6 weeks to quantify response and adjust field values; running time checks; and visually inspecting each deployment following protocol. As previously described [36,37,38], the adjusted MicroPEM nephelometer data were visually validated, checking if the data contained significantly negative readings for extended time periods, improbable plateaus of high values, or “stair-step” increases and decreases in the raw baseline. Only visually valid PM_2.5_ data were retained for the study. As previously done for GRAPHS analyses, CO and PM_2.5_ exposures were based on the first 48 h of each 72 h deployment to avoid situations where battery issues or pick-up schedules may have missed a cooking episode on the final day of deployment [8,36,37].

### 2.3. Stove Interventions 

GRAPHS assigned communities (clusters) to one of two stove interventions or control [36]. Participants in the LPG arm received a two-burner LPG stove (Ghana Cylinder Company, Accra), two 14.5-kilogram LPG cylinders and monthly gas refills as needed, beginning after randomization and through child age 12 months. Additional fuel was provided as needed. Participants in the improved biomass arm received two single-burner BioLite HomeStove forced draft wood fuel cookstoves (BioLite Inc, Brooklyn, NY, USA). The improved combustion efficiency stove reduces emissions via geometry and a thermoelectric-powered fan, which improve heat transfer efficiency and combustion efficiency, respectively. Women randomized to the control arm continued using their traditional, open fire stove. Fieldworkers visited each household weekly to encourage stove use and repair stoves as necessary. 

### 2.4. Telomere Length Measurement 

Procedures for cord blood sampling, mononuclear cell isolation and DNA extraction in this cohort have been described elsewhere [8]. Briefly, 10 mL of cord blood was collected into BD vacutainer heparinized tubes immediately after delivery and transported to the KHRC Clinical Laboratory on IsoRack cool packs (Iso Therm-System, Hamburg, Germany). Upon arrival at the laboratory, CBMCs were isolated by density gradient centrifugation, and pellets were stored at –80 °C until analyses.

Mean TL was determined by duplex quantitative PCR (qPCR) to compare the relative amplification of the telomere repeat copy number with single gene (albumin) copy number. DNA was extracted from CBMC pellets, and samples were normalized to 2 ng/µL and concentrations were confirmed using PicoGreen quantification prior to amplification. The primers for qPCR of TL were: Telc 5′-TGT TAG GTA TCC CTA TCC CTA TCC CTA TCC CTA TCC CTA ACA-3′ and Telg 5′-ACA CTA AGG TTT GGG TTT GGG TTT GGG TTT GGG TTA GTG T-3′. Additionally, iQ SYBR Green Supermix, which contains an antibody-mediated hot-start iTaq DNA polymerase as well as a passive reference dye fluorescein, was used. Samples were amplified per previously established protocols [39,40]. Each sample was run in triplicate, and a pooled quality control sample was run on each plate. The coefficient of variation (CV) for triplicate samples was calculated, and a threshold of 0.13, determined using the inter-quartile range, was used for inclusion in the analysis. The telomere/single copy gene ratio (T/S ratio) was calculated as the ratio of telomere copy number relative to albumin copy number, both of which were estimated by the Bio-Rad software using the study-specific standard curve (Cq = slope × Log10(Sq) + intercept). We divided the per plate T/S ratio of the pooled DNA sample by the average T/S ratio across plates for all pooled samples to obtain a normalizing factor. We then divided samples on a given plate by this plate-specific normalizing factor to adjust for potential batch effects [41] and to determine the CBMC relative telomere length (rTL) for analyses. 

### 2.5. Resting Blood Pressure at Child Age Four Years 

Resting blood pressure was measured at child age four years per protocol using the Omron 5 Series oscillometric blood pressure monitor with appropriately fitted cuff. Briefly, the child was seated undisturbed for 10 minutes and then underwent two blood pressure measurements separated by five minutes. Systolic and diastolic blood pressure were recorded from each resting blood pressure measurement and averaged. 

### 2.6. Covariates 

Data on maternal age, maternal education and ethnicity as well as secondhand smoke exposure were obtained through questionnaire at enrolment. Household assets were queried on enrolment and were used to generate a wealth index to describe household-level socioeconomic status relative to other households in the study [42]. Maternal weight and height were measured at enrolment to the nearest 0.1 kilogram and 0.1 centimeter, respectively, and were used to calculate maternal BMI. Gestational age was determined using the date of delivery and the previously established ultrasound estimated date of delivery [43]. CBMC storage time was determined as the difference between the dates of telomere analyses minus date of birth. Child sex was recorded at delivery. 

### 2.7. Statistical Analysis 

The primary analytic cohort for these analyses included the *n* = 97 children with available data on prenatal HAP exposure, cookstove intervention arm, CBMC rTL and age four blood pressure. A subset of these mother–infant pairs (*n* = 60) additionally had prenatal PM_2.5_ exposure data. For all analyses, we natural log-transformed CBMC rTL due to its right-skewed distribution.

We first analyzed associations between prenatal CO and PM_2.5_ exposures, considered separately, and CBMC rTL using univariate and multivariable linear regression. We then explored associations between GRAPHS cookstove intervention arm and CBMC rTL again using univariate and multivariable linear regression. Given that rTL was natural log-transformed, we expressed the β coefficients from these models as percentage change in rTL. Finally, we employed linear regression to examine associations between CBMC rTL and resting blood pressure at child age four. β coefficients were expressed as unit change in blood pressure per percentage change in rTL. Multivariable models adjusted for child sex, maternal education, maternal BMI, age and ethnicity, gestational age at delivery and CBMC storage time. 

We also performed a series of sensitivity models. First, we repeated the analyses between prenatal CO and PM_2.5_ exposures and cookstove intervention arm and CBMC relative TL using the larger (*n* = 138 for CO and *n* = 81 for PM_2.5_) mother–infant pair sample. This larger cohort included 41 mother–infant pairs without age 4 blood pressure data. Second, we considered multipollutant models with both CO and PM_2.5_ in the model and additional adjustment for second-hand tobacco smoke exposure or household-level socioeconomic status (wealth index). Third, the association between rTL and resting BP may vary based on child sex. Therefore, using stratified analysis, we explored child sex as a potential effect modifier.

## 3. Results

One hundred and fifty-seven CBMC pellets were available for DNA extraction. In *n* = 18 samples, insufficient DNA was extracted, leaving *n* = 139 samples for telomere analyses. Of these, *n* = 138 had valid CO data, and *n* = 60 had valid PM_2.5_ data. Age four blood pressure data were available for *n* = 97. 

Participant characteristics are summarized in Table 1. 

Approximately half of the infants were girls (*n* = 49, 50.5%). The majority of mothers had at least primary-level education (*n* = 59, 60.8%). The median maternal age at enrolment was 26 years (IQR 22–33), and the median gestational age at delivery was 39.7 weeks (IQR 39.0–40.6). The median prenatal maternal CO was 0.85 ppm (IQR 0.49–1.42), and the median prenatal PM_2.5_ in those with available data was 58.3 µg/m^3^ (IQR 37.3–84.3, *n* = 60). A total of 24 (24.7%), 27 (27.8%) and 46 (47.4%) mothers belonged to the LPG, improved biomass and open fire (control) arms, respectively. In the overall cohort, the median plate-pool normalized CBMC rTL was 0.68 (IQR 0.55–0.88). The median systolic blood pressure (SBP) was 91 mmHg (IQR 86–97), and the median diastolic blood pressure (DBP) was 64 mmHg (IQR 58-70.5). Participants in the larger telomere cohort (*n* = 138) were similar in baseline characteristics (Table S1).

### 3.1. Exposure-Response Associations between Prenatal CO and CBMC Relative TL

Univariate and multivariable models did not demonstrate an association between prenatal average CO exposure and percentage change in CBMC rTL (univariate model: β= −1.0% change (95% CI −6.8, 6.2), *p* = 0.82; multivariable model: β = −3.0% change (95% CI −9.5, 4.1), *p* = 0.46) per 1 ppm increase in average prenatal CO (Table 2). Sensitivity models additionally adjusting for second-hand smoke exposure and household SES did not change these findings (Table S2). Sensitivity analysis using the larger cohort (*n* = 138) similarly did not suggest an association between prenatal average CO exposure and percentage change in CBMC relative TL (multivariable model β= −2.0% (95% CI −8.6, 4.1), *p* = 0.47) per 1 ppm increase in average prenatal CO (Table S3).

### 3.2. Exposure-Response Associations between Prenatal PM_2.5_ and CBMC Relative TL

A 10 µg/m^3^ increase in average prenatal PM_2.5_ was associated with a 4.9% reduction ((95% CI −8.6, −0.4), *p* = 0.03) in CBMC rTL at birth in all children, after adjusting for maternal education, BMI, age, ethnicity, and child sex and CBMC storage time (Table 2). In sex-stratified analysis, a 10 µg/m^3^ increase in average prenatal PM_2.5_ was associated with a 7.4% decrease in rTL ((95% CI −13.8, −0.50), *p* = 0.04) in male newborns only. There was no evidence for an association in female newborns. Sensitivity models additionally adjusting for second-hand smoke and household SES did not substantively change these findings (Table S2). Sensitivity analyses in the larger cohort (*n* = 81) found that a 10 µg/m^3^ increase in PM_2.5_ was associated with a 3.9% reduction in relative TL ((95% CI −7.7, −0.7), *p* = 0.02) following adjustment for maternal education, BMI, age, ethnicity, and child sex and CBMC storage time (Table S3).

A multipollutant model including both CO and PM_2.5_ similarly found that an increase in prenatal PM_2.5_ but not CO was associated with a reduction in CBMC rTL (CO β = −9.5% change (95% CI −30.2, 16.2), *p* = 0.41 per 1ppm increase; PM_2.5_ β = −3.9% change (95% CI −8.6, −0.10), *p* = 0.048 per 10 µg/m^3^ increase). 

### 3.3. Effect of Cookstove Intervention on CBMC Relative TL

For this subset of GRAPHS participants, the average prenatal PM_2.5_ exposure in LPG, improved biomass and control arms was 42.7 µg/m^3^, 57.1 µg/m^3^ and 79.2 µg/m^3^, respectively. The average prenatal CO exposure in LPG, improved biomass and control arms was 0.73 ppm, 0.97 ppm, 1.66 ppm, respectively. In exploratory analyses of the effect of cookstove intervention on newborn CBMC rTL, newborns of mothers randomized to the LPG arm had, on average, 45% longer relative TL compared with newborns of mothers randomized to the control arm (β = 44.8% (95% CI 9.4, 89.6), *p* < 0.01). This effect was stronger following adjustment for maternal education, BMI, age, and ethnicity and child sex and CBMC storage time (β = 55.3% (95% CI 16.2, 109.6), *p* < 0.01) (Table 2). No statistically significant association was seen between the improved combustion efficiency biomass stove and CBMC rTL as compared with the control arm. Sensitivity analyses in the larger cohort suggest a smaller effect, with newborns of mothers randomized to the LPG arm having, on average, 36.3% longer rTL compared with newborns of mothers randomized to the control arm (β = 36.3% (95% CI 7.3, 75.1), *p* = 0.01).

### 3.4. Exposure-Response Relationship between CBMC Relative TL and Blood Pressure at Age Four Years 

In linear regression models, CBMC rTL was inversely associated with systolic BP (SBP) at age four years. A 10% decrease in CBMC rTL was associated with a 0.34 mmHg increase in systolic BP (β = 0.34 (95% CI 0.09, 0.68), *p* = 0.04) (Table 3). Following adjustment for child sex, maternal education, maternal BMI, maternal age, ethnicity, gestational age at delivery and storage time, a 10% decrease in CBMC rTL was associated with a 0.35 mmHg increase in systolic BP (β = 0.35 (95% CI 0.001, 0.71), *p* = 0.05). When the results were stratified by child sex, a 10% decrease in CBMC rTL was associated with a 0.73 mmHg increase in systolic BP (β = 0.73 (95% CI 0.22, 1.24), *p* < 0.01) in males only; no association was seen in females. CBMC rTL was not associated with diastolic BP in all children or in sex-specific analyses. 

## 4. Discussion

These data extend the growing literature linking prenatal air pollution exposure to cord blood TL to now include prenatal HAP exposure. We examined the association between prenatal HAP exposure, as indexed by both CO and PM_2.5_, on CBMC TL and how TL at birth may be associated with future blood pressure. Our data suggest that increasing prenatal PM_2.5_, and not CO, exposure was associated with shorter TL at birth. Exploratory analyses suggest that a prenatal clean fuel intervention may ameliorate this effect. Further, our data support the concept of TL at birth as a biomarker of environmental risk and demonstrate associations between TL at birth and age four resting blood pressure, a clinical marker of cardiovascular health. Taken together, our findings suggest that TL as a biomarker of exposure and BP may be a mediator of the association between HAP exposure and HAP-associated adverse cardiovascular health. However, our sample was too small to perform a formal mediation analysis. 

Previous studies have demonstrated associations between prenatal ambient air pollution exposure and newborn TL, with mixed results. In the context of studies that have demonstrated a similar direction of association—namely, that increased prenatal exposure over pregnancy reduces telomere length at birth—we note that our results show a smaller magnitude of effect. For example, work in the NYC-based PRISM cohort found that a cumulative 1 μg/m^3^ increase in prenatal PM_2.5_ exposure was on average associated with a 0.29, or 12%, reduction in plate-pool normalized rTL in a cohort with an average rTL of 2.4 [21]. ENVIR*ON*AGE reported an overall 8.8% reduction per 5 μg/m^3^ increase over pregnancy [20]. By contrast, our results suggest a 5% reduction in relative TL per 10 μg/m^3^ increase in average prenatal PM_2.5_ exposure. We note that our PM_2.5_ exposure assessment measured 72-h personal PM_2.5_ at one time-point prenatally, while both the PRISM and ENVIR*ON*AGE studies employed residence-specific daily PM_2.5_ estimates over gestation. 

The burning of solid fuels results in a complex mixture of pollutants. Our study measured PM_2.5_, a complex mixture including primary and secondary combustion related particles and re-suspended dust, and CO, allowing us to explore the effect of each pollutant individually and in co-pollutant sensitivity models. These data suggest that PM_2.5_ exposure rather than CO is associated with reductions in newborn TL. Work by Song et al. leveraging a birth cohort from Wuhan, China, and PM_2.5_ and CO estimates derived from spatial–temporal land use regression models found no association with overall pollutant exposures but did, in contrast, find that both increased third trimester average PM_2.5_ and that CO exposures were independently associated with reductions in newborn TL [22]. The associations with CO did not persist in multipollutant models. Of note, the overall air pollution levels in the Wuhan cohort were more similar to ours (for example, third trimester PM_2.5_ average 69.1 μg/m^3^ (20.04SD) and CO average 988 μg/m^3^ (170.5)), as was the cohort average cord blood telomere length (T/S ratio median 0.74, IQR 0.56, 0.95). 

Two studies have reported associations between HAP exposure and TL, and these studies are limited to adults. For example, a study in *n* = 137 Chinese adults who cooked primarily with wood-based cookstoves measured personal PM_2.5_ exposures for 48-hours prior to sampling buccal cell DNA, with subsequent analysis for telomere length. Filters were also analyzed for black carbon (BC) content. These analyses found that increased PM_2.5_ and BC were inversely associated with buccal cell TL [26]. A second study recruited *n* = 137 Chinese adults from two Beijing Hospitals and leveraged questionnaire data on solid fuel use gathered over three decades. Peripheral venous blood was collected at enrolment and leukocyte TL measured subsequently. The authors found that solid fuel use for three decades was associated with shorter peripheral blood leukocyte TL compared with non-use of solid fuels [25]. 

While no study has examined the effect of a cookstove intervention to ameliorate the effects of HAP on TL, one prior study evaluated the impact of environmental policies or interventions to improve air quality on health outcomes. Perera et al. evaluated the impact of closure of local coal plants in Chongqing Municipality, Tongliang County, China, on telomere length [44]. This study found that infants conceived and born after coal plant closure had on average longer TL as compared with those conceived and born before coal plant closure. In the present study, newborns of women randomized to use LPG had on average a 36.5 µg/m^3^ (54%) reduction in prenatal PM_2.5_ exposure as compared with newborns of women who continued to cook over the traditional open fire stove. Though both groups had exposures above the WHO indoor air quality guidelines [45], newborns of mothers in the LPG arm had on average 55% longer telomeres, suggesting an exposure–response relationship at even these higher levels of air pollution. 

The mechanisms underlying the health effects of environmental exposures, including HAP, on telomere length are not fully understood. Oxidative stress and chronic inflammation have been proposed as the two principal mechanisms. HAP-induced oxidative stress leads to increased energy demand and thus increased oxidative phosphorylation, which in turn releases more free radicals, damaging intracellular constituents including nuclear and mitochondrial DNA. Alterations in mitochondrial DNA could in turn influence cellular ageing and telomere length via defects in oxidative phosphorylation [46]. Oxidative stress also leads to the influx of inflammatory cells to the site of oxidative damage, leading to more free radical release and tissue damage [47]. Supporting this, recent work suggests that maternal antioxidant intake during pregnancy may ameliorate the effects of prenatal ambient air pollution exposure on newborn telomere length [21]. 

Our findings add to the growing literature linking telomere to indicators of cardiovascular health [35,48,49,50]. Specifically, we find that shorter telomere length at birth is associated with higher resting systolic blood pressure at age four years. Sex-specific effects were limited by sample size but suggested that while all children are affected, boys may be especially vulnerable. This sex-differential effect could be explained by the fact that TL on average is longer in females compared with age-matched males [23,24,51], as is the case in this cohort (*p* < 0.01). Supporting evidence comes from O’Donnell et al. who found that leukocyte TL was associated with carotid artery intima–media thickness in men but not in women [49]. Several studies have shown an association between telomere shortening and higher risk of hypertension in adults [52,53,54]. However, studies on TL and markers of cardiovascular health in childhood are few and show mixed results. In a longitudinal birth cohort study in Sydney, Australia, shorter TL at age three years was associated with greater carotid artery intima–media thickness at eight years of age [55]. In contrast, Nguyen et al. found no association between leukocyte TL and carotid intima–media thickness or carotid–femoral pulse wave velocity in Australian children aged 11 years [56]. Of note, the study by Nguyen et al. employed a cross-sectional design, with TL and vascular endpoints measured at the same timepoint. Our findings support prior work suggesting that TL at birth may be a marker of environmental risk and further suggest that this TL setpoint at birth may predict subsequent markers of cardiovascular health. 

We note several strengths of our study. We leveraged GRAPHS, a cookstove intervention study that randomized pregnant women to one of two cookstove interventions, including a clean cookstove, and performed repeated personal exposure measurements over the prenatal period. In a subset of mother–infant dyads, we collected CBMCs at birth, with subsequent analysis for telomere allowing us to examine exposure–response relationships of two key HAP pollutants and to explore the potential ameliorating effect of cookstove interventions. Our prospective, longitudinal follow-up of the cohort with protocolized assessment of resting blood pressure at age four allowed us to extend these analyses to understand associations between telomere length at birth and early childhood blood pressure, a marker of cardiovascular health. We followed rigorous and standardized protocols for exposure assessment, TL measurement and blood pressure assessment. Our well-described cohort allowed us to control for important covariates. 

We also note a number of limitations. While we control for a number of important confounders, we note the possibility for residual confounding, most notably from diet and other environmental exposures. Due to logistical constraints, we were unable to perform multiple PM_2.5_ measurements across pregnancy. We measured one marker of cardiovascular health at one time point (child age four years). Future studies should perform longitudinal measurement of resting blood pressure and should consider a more precise measure of blood pressure, such as ambulatory blood pressure monitoring [57]. Additional measures such as carotid artery intima–media thickness, pulse wave velocity and insulin resistance in the pediatric population would provide a more complete picture of cardiovascular health. Our subsample of the GRAPHS cohort included only children who were born in a health facility, thus potentially selecting children whose mothers were better educated or who were from households with better socio-economic status compared with the rest of the GRAPHS cohort. However, children included in the current analysis were largely comparable with the parent GRAPHS cohort in terms of baseline characteristics. Last, our results are limited by our small sample size; thus, a formal mediation analysis replication in a larger cohort is important. 

In summary, shorter TL at birth may reflect a “set-point” for telomere length across the life course [51]. We find that increased prenatal HAP exposure, specifically PM_2.5_, is associated with shorter TL at birth; exploratory analyses suggest a clean fuel intervention may ameliorate this effect. Further, we find that TL at birth is associated with child systolic blood pressure, an important marker of cardiovascular health. These data warrant replication and confirmation because an elucidation of molecular pathways that underlie associations between prenatal HAP exposure and early life health can begin to build an evidence base for biomarkers for future preventive efforts.

## Figures and Tables

**Table 1:** Participant Characteristics.

Continuous Variables (Median, Interquartile Range)	All (*n* = 97)	Male (*n* = 48)	Female (*n* = 49)
Cord blood mononuclear cell telomere length *	0.68 (0.55–0.88)	0.63 (0.48–0.81)	0.71 (0.59–0.98)
CBMC storage time (years)	2.57 (2.41–2.84)	2.62 (2.41–2.84)	2.57 (2.40–2.78)
Prenatal household air pollution exposure **			
Carbon monoxide (CO), ppm	0.85 (0.49, 1.42)	0.83 (40, 1.27)	0.94 (0.60, 1.54)
Fine particulate matter (PM_2.5_), μg/m^3^	58.3 (37.3, 84.3)	50.1 (35.8, 85.3)	60.8 (41.3, 79.0)
Systolic blood pressure at age four years^Ω^, mmHg	91 (86, 97)	91 (86.9, 95)	90.5 (84, 97)
Diastolic blood pressure at age four years^Ω^, mmHg	64 (58, 70.5)	62.8 (58, 69.8)	65 (58, 71)
Gestational age at delivery, weeks	39.7 (39.0, 40.6)	39.7 (39.0, 40.3)	39.9 (39.1, 40.7)
Maternal characteristics			
Age, years	26 (22, 33)	27 (23, 34)	24.5 (21, 32)
Body mass index, Kg/m^2^	22.5 (21.1, 24.1)	23.0 (21.6, 24.6)	22.2 (20.8, 23.7)
Categorical variables (*n*, %)			
Cookstove intervention arm			
Control	46 (47.4)	21 (43.8)	25 (51)
Improved biomass	27 (27.8)	14 (29.2)	13 (26.5)
Liquefied petroleum gas	24 (24.7)	13 (27.1)	11 (22.4)
Maternal education			
None	38 (39.2)	18 (37.5)	20 (40.8)
Primary school or higher	59 (60.8)	30 (62.5)	29 (59.2)
Ethnicity			
1	28 (28.9)	14 (29.2)	14 (28.6)
2	19 (19.6)	10 (20.8)	9 (18.4)
3	26 (26.8)	13 (27.1)	13 (26.5)
4 (other)	24 (24.7)	11 (22.9)	13 (26.5)

* T/S ratio normalized against plate pool average. ** Personal exposure to household air pollution assessed by CO (*n* = 97) in parts per million and PM_2.5_ (*n* = 60, Male *n* =32, Female *n* = 28) in ug/m^3^. Ω Resting systolic and diastolic blood pressure measured at child age four years twice after rest per protocol using the Omron automated BP cuff and then averaged.

**Table 2:** Percentage change in cord blood leukocyte telomere length per unit increase in household air pollution (HAP) exposure or cookstove intervention arm as compared with control: linear regression.

HAP Exposure **	*n*	Univariate Model		Multivariable Model *	
		β (95% CI)	*p*-Value	β (95% CI)	*p*-Value
Average prenatal CO	97	−1.0 (−6.8, 6.2)	0.82	−3.0 (−9.5, 4.1)	0.46
Average prenatal PM_2.5_	60	−3.9 (−7.7, 0.5)	0.08	−4.9 (−8.6, −0.4)	0.03
Sex-specific Associations					
Boys					
Average prenatal CO	48	−0.3 (−11.2, 13.3)	0.96	−4.3 (−15.6, 8.6)	0.49
Average prenatal PM_2.5_	32	−5.2 (−11.1, 1.1)	0.10	−7.4 (−13.8, −0.5)	0.04
Girls					
Average prenatal CO	49	−1.8 (−9.7, 6.7)	0.66	−4.5 (−13.2, 5.1)	0.34
Average prenatal PM_2.5_	28	−3.2 (−9.0, 3.0)	0.29	−3.4 (−11, 4.9)	0.39
Cookstove Intervention Arm					
Control (open fire)	46	Ref	–	Ref	–
Improved biomass stove	27	23.4 (–4.9, 61.6)	0.12	25.9 (−4.9, 68.2)	0.11
LPG	24	44.8 (9.4, 89.6)	<.01	55.3 (16.2, 109.6)	<.01

* Models are adjusted for child sex, maternal education, BMI and age, ethnicity and CBMC storage time. ** CO models are interpreted as percentage change in CBMC telomere length per 1ppm increase in average prenatal exposure. PM_2.5_ models are interpreted as percentage change in CBMC telomere length per 10ug/m^3^ increase in average prenatal exposure.

**Table 3:** Change in age four resting blood pressure per 10% increase in cord blood mononuclear cell relative telomere length for all children and by sex: linear regression (*n* = 97).

Resting Blood Pressure	*n*	Univariate Model		Multivariable Model	
		β (95% CI)	*p*-Value	β (95% CI)	*p*-Value
All Children *					
Systolic blood pressure, mmHg	97	−0.34 (−0.68, −0.09)	0.04	−0.35 (−0.71, −0.001)	0.05
Diastolic blood pressure, mmHg	97	−0.16 (−0.49, 0.17)	0.35	−0.18 (−0.54, 0.17)	0.31
Sex-specific Associations **					
Boys					
Systolic blood pressure, mmHg	48	−0.61 (−1.09, −0.12)	0.01	−0.73 (−1.24, −0.22)	<.01
Diastolic blood pressure, mmHg	48	−0.24 (−0.75, 0.26)	0.34	−0.28 (−0.84, 0.29)	0.33
Girls					
Systolic blood pressure, mmHg	49	−0.09 (−0.58, 0.40)	0.71	−0.09 (−0.62, 0.44)	0.74
Diastolic blood pressure, mmHg	49	−0.13 (−0.60, 0.35)	0.59	−0.23 (−0.32, 0.32)	0.41

* Multivariable models in all children adjusted for child sex, maternal education, maternal BMI, maternal age, ethnicity, gestational age at delivery and CBMC storage time. ** Sex-specific models stratified by child sex. Multivariable models adjusted for maternal education, maternal BMI, maternal age, ethnicity, gestational age at delivery and CBMC storage time.

## Data Availability

The data presented in this study are available on request from the corresponding author. The data are not publicly available due to institutional and national data sharing restrictions.

## Supplementary Materials

The following are available online at https://www.mdpi.com/article/10.3390/toxics9070169/s1, Table S1: participant characteristics of larger telomere cohort, Table S2: sensitivity models examining the association between prenatal household air pollution measures as indexed by maternal personal CO and PM_2.5_ measurements and GRAPHS study arm, considered separately, and log-transformed cord blood leukocyte telomere length: linear regression, Table S3: Larger cohort (*n* = 138) association between prenatal household measure as indexed by maternal personal CO and PM_2.5_ measurements and GRAPHS study arm, considered separately, and log-transformed cord blood leukocyte telomere length: linear regression.
