# Supplementary material for: Prenatal Household Air Pollution Exposure, Cord Blood Mononuclear Cell Telomere Length and Age Four Blood Pressure: Evidence from a Ghanaian Pregnancy Cohort
Source: Toxics. 2021 Jul 14;9(7):169. doi: 10.3390/toxics9070169 (PMC8309911; doi:10.3390/toxics9070169)
Supplement: Supplementary file 1 [file toxics-09-00169-s001.zip › toxics-1268980-supplementary.pdf]

toxics

MDPI

Article

Supplementary Materials: Prenatal Household Air Pollution Exposure, Cord Blood Mono-nuclear Cell Telomere Length and Age Four Blood Pressure: Evidence from A Ghanaian Pregnancy Cohort

Seyram Kaali, Darby Jack, Jones Opoku-Mensah, Tessa Bloomquist, Joseph Aanaro, Ashlinn Quinn, Ellen Abrafi Boamah-Kaali, Patrick Kinney, Mohammed Nuhu Mujtaba, Oscar Agyei, Abena Konadu Yawson, Samuel Osei-Owusu, Rupert Delimini, Blair Wylie, Kenneth Ayuurebobi Ae-Ngibise, Andrea Baccarelli, Seth Owusu-Agyei, Steven N Chillrud, Kwaku Poku Asante and Alison Lee

Table S1. Participant Characteristics of larger telomere cohort.

Continuous Variables (Median, Interquartile Range)	All (*n* = 138)		Male (*n* = 68)		Female (*n* = 70)	
Cord blood mononuclear cell (CBMC) telomere length*	0.68	0.56, 0.87	0.66	0.49, 0.81	0.69	0.59, 0.99
CBMC storage time (years)	2.6	2.41, 2.83	2.63	2.41, 2.84	2.57	2.41, 2.78
Prenatal household air pollution exposure**						
Carbon monoxide (CO), ppm	0.85	0.51, 1.45	0.79	0.41, 1.34	1.01	0.68, 1.54
Fine particulate matter ($PM_{2.5}$), ug/m^3	57	38.4, 83.4	50.6	37.3, 82.2	61.3	42.2, 84
Gestational Age at Delivery (weeks)	39.7	39, 40.7	39.7	39, 40.5	39.7	39, 40.7
Maternal Characteristics						
Age (years)	25	22, 33	24	20, 32	27	23, 33
Body Mass Index (kg/m^2)	22.7	21.1, 24.2	23.1	21.6, 24.6	22.3	20.8, 23.8
Categorical Variables (*n*, %)						
Cookstove intervention arm						
Control	63	45.7%	30	44.1%	33	47.1%
Improved Biomass	39	28.3%	20	29.4%	19	27.1%
Liquefied Petroleum Gas	36	26.1%	18	26.5%	18	25.7%
Maternal education						
None	52	37.7%	24	35.3%	28	40%
Primary school or higher	86	62.3%	44	64.7%	42	60%
Ethnicity						
1	36	26.1%	20	29.4%	16	22.9%
2	24	17.4%	12	17.6%	12	17.1%
3	40	29.0%	19	27.9%	21	30%
4 (other)	38	27.6%	17	25%	21	30%

*T/S ratio normalized against plate pool average, **Personal exposure to household air pollution assessed by CO (*n* = 138) in parts per million and $PM_{2.5}$ (*n* = 81, Male *n* = 44, Female *n* = 37) in ug/m^3.

Table S2. Sensitivity models examining the association between prenatal household air pollution measures as indexed by maternal personal CO and $PM_{2.5}$ measurements and GRAPHS study arm, considered separately, and log-transformed cord blood leukocyte telomere length: linear regression .

HAP Exposure	Multipollutant Model		+ Second-Hand Smoke		+ Household SES	
	β (95% CI)	*p*-Value	β (95% CI)	*p*-Value	β (95% CI)	*p*-Value
Average prenatal CO	−9.5 (−30.2, 16.2)	0.41	−1.0 (−18.9, 22.1)	0.92	0.1 (−19.7, 19.7)	0.99
Average prenatal $PM_{2.5}$	−3.9 (−8.6, −0.10)	0.048	−5.8 (−9.5, −1.0)	0.02	−5.8 (−9.5, −1.0)	0.02
Cookstove Intervention Arm						
Control (open fire)	–	–	Ref	–	Ref	–
Improved biomass stove	–	–	13.9 (−12.2, 49.2)	0.32	18.5 (−9.5, 56.8)	0.21
LPG	–	–	58.1 (15.6, 216)	0.003	71.6 (31.0, 124.8)	<.001

*All models are adjusted for child sex, maternal education, maternal BMI, maternal age, ethnicity and CBMC storage time. CO models are interpreted as per 1ppm change. $PM_{2.5}$ models are interpreted as per 10ug/m^3 change.

Table S3. Larger cohort (n = 138) association between prenatal household measure as indexed by maternal personal CO and $PM_{2.5}$ measurements and GRAPHS study arm, considered separately, and log-transformed cord blood leukocyte telomere length: linear regression

HAP Exposure	*n*	Univariate Model		Multivariable Model	
		β (95% CI)	*p*-Value	β (95% CI)	*p*-Value
Average prenatal CO	138	−1.0 (−7.6, 5.1)	0.70	−2.0 (−8.6, 4.1)	0.47
Average prenatal $PM_{2.5}$	81	−3.9 (−6.8, 0.1)	0.054	−3.9 (−7.7, −0.70)	0.02
Sex-specific Associations					
Boys					
Average prenatal CO	68	−0.7 (−11.3, 11.1)	0.90	−3.7 (−14.7, 8.7)	0.53
Average prenatal $PM_{2.5}$	44	−4.2 (−9.3, 1.2)	0.12	−5.4 (−11.5, 1.1)	0.10
Girls					
Average prenatal CO	70	−2.3 (−10, 6)	0.57	−4.6 (−12.4, 3.9)	0.27
Average prenatal $PM_{2.5}$	37	−4.1 (−9.2, 1.3)	0.13	−3.3 (8.9, 2.7)	0.26
Cookstove Intervention Arm					
Control (open fire)	63	Ref	--	Ref	--
Improved biomass stove	39	10.5 (-12.2, 39.1)	0.37	9.4 (−13.1, 37.7)	0.46
LPG	36	29.7 (2.0, 63.2)	0.03	36.3 (7.3, 75.1)	0.01

*Models are adjusted for child sex, maternal education, maternal BMI, maternal age, ethnicity and CBMC storage time. CO models are interpreted as percent change in CBMC telomere length per 1ppm increase in CO. $PM_{2.5}$ models are interpreted as percent change in relative telomere length per 10ug/m^3 increase in $PM_{2.5}$.
